# Supplementary material for: Computational models of category-selective brain regions enable high-throughput tests of selectivity
Source: Nat Commun. 2021 Sep 20;12:5540. doi: 10.1038/s41467-021-25409-6 (PMC8452636; doi:10.1038/s41467-021-25409-6)
Supplement: Supplementary file 1 — Supplementary Information [file 41467_2021_25409_MOESM1_ESM.pdf]

Computational models of category-selective brain regions enable high-throughput tests of selectivity

N. Apurva Ratan Murty*, Pouya Bashivan*, Alex Abate, James J DiCarlo, and Nancy Kanwisher

Supplemental Information

Supplemental Figures

S1. fMRI data reliability as a function of number image repetitions

S2. Observed responses in the dynamic localizer and event-related paradigm

S3. Screening computational models based on pooled metrics

S4. DNN-based models generalize across stimuli and subjects

S5. Screening computational models based on population-level metrics

S6. Observed and model-predicted RDMs (for 2 representative models) for each subject and fROI

S7. Sub-sampled stimuli from the top 100,000 images that the models predict most strongly activate the human FFA

S8. Sub-sampled stimuli from the top 100,000 images that the models predict most strongly activate the human EBA

S9. Sub-sampled stimuli from the top 100,000 images that the models predict most strongly activate the human PPA

S10. Sub-sampled stimuli from the top 100,000 images (excluding stimuli from the VGGFace database) that the models predict most strongly activate the human FFA

S11. Sub-sampled stimuli from the top 100,000 images (excluding stimuli from the VGGFace database) that the models predict most strongly activate the human PPA

S12. Negative control analysis based on simulating the fMRI experiment on conv-5 units of Alexnet

S13. Schematic outline of the data-handling procedures for an example region (left-EBA)

Supplemental Table

S1. Summary of all the models evaluated in the current study with the model layer with the best cross-validated accuracy at predicting the responses to images in the different fROIs

References

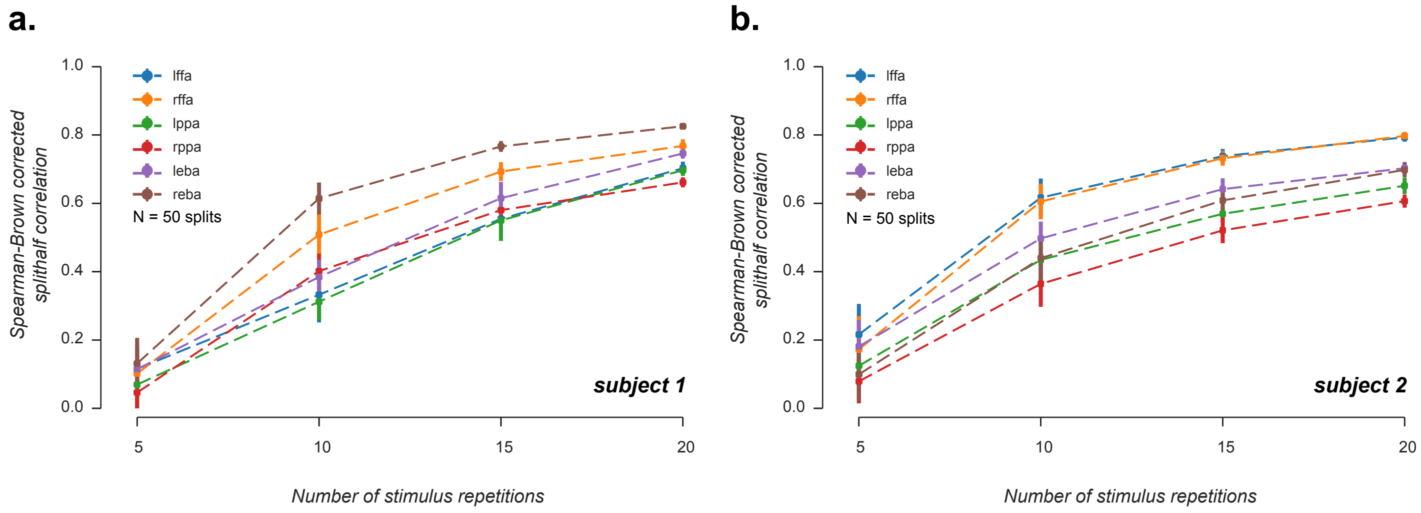

Supplementary Figure 1. Voxel-wise fMRI reliability as a function of number of repetitions per image. Here we determined the number of repetitions per image required to obtain reliable image-wise beta estimates in an event-related fMRI experiment. Data reliability was estimated by taking 50 random subsamples of 5, 10, 15 or 20 trials. For each subsample, the data were further split into 2 random groups and re-analyzed to obtain the fMRI beta estimates for each of the 185 images. The reliability of the data was measured for each vertex as the Spearman-Brown corrected splithalf correlation between the 2 splits and then averaged across the 50 random subsamples.

a. Data reliability (y-axis) as a function of number of splits (x-axis) for Subject 1. The lines indicate the median vertex-wise reliability in a given fROI over 50 random splits of the data and the error-bars indicate the standard deviation across the 50 splits. Based on this, we decided to include 20 repetitions per image for all participants. Source data are provided as a Source Data file

b. Same as **a.** but for Subject 2**.** Source data are provided as a Source Data file

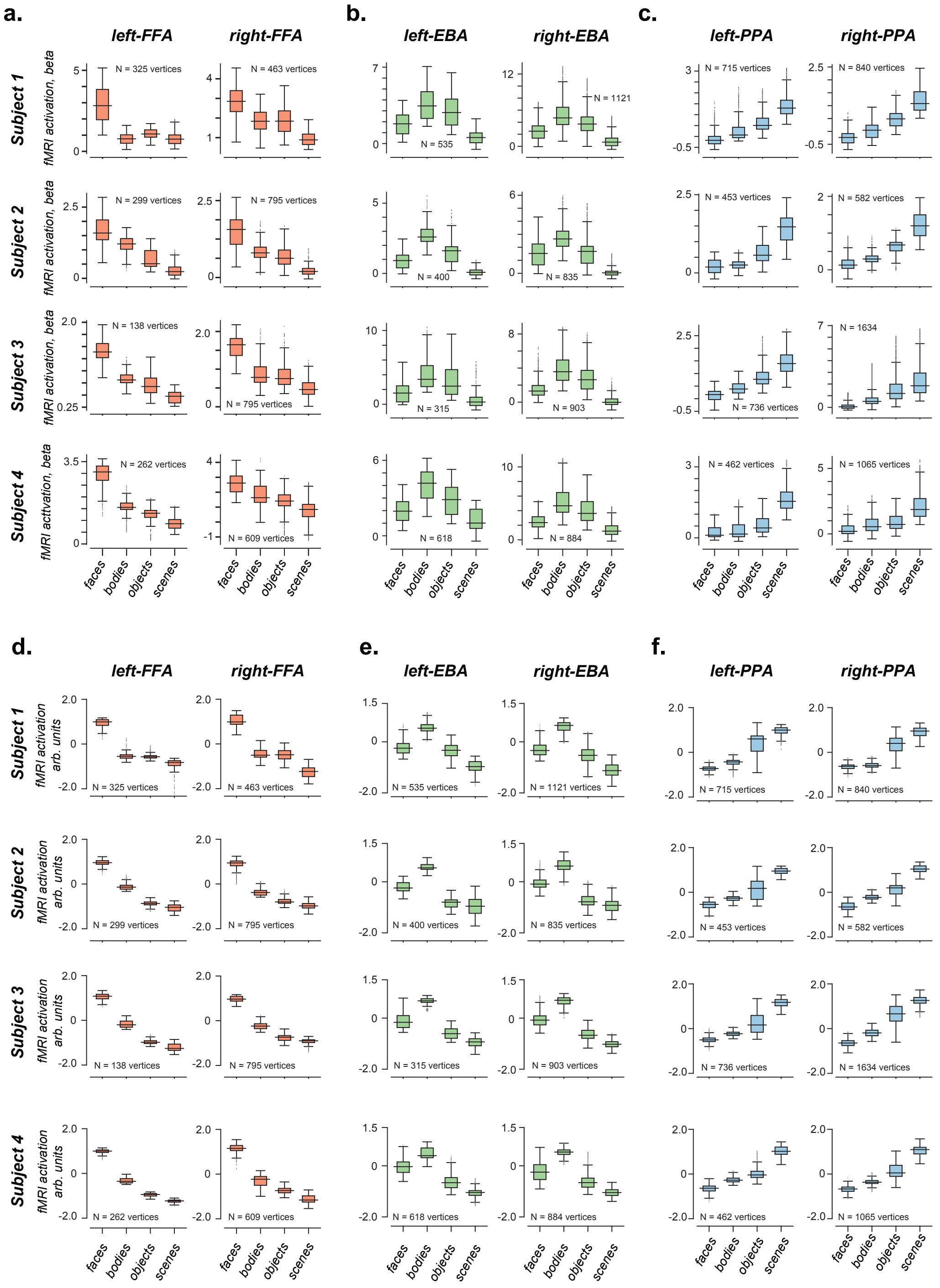

Supplementary Figure 2. Observed responses averaged across stimulus categories in the dynamic localizer and the event-related experiment.

a. Boxplots indicating the median (line in the middle) observed fMRI beta parameters (y-axis) for each of the four categories for the left and right FFA across vertices. The number of vertices within each region are included in the figure. The boxes indicate the interquartile range, and the ends of the lines indicate the minimum and maximum values. Outliers, if any, are shown using gray dots. Note – these data are not independent (see **d-f** for independent data) as the fROIs were determined based on the same data. Source data are provided as a Source Data file

b-c. Same as **a.** but for the EBA and the PPA. Source data are provided as a Source Data file

d. Boxplots indicating the median observed fMRI responses (normalized across sessions – see Methods) (y-axis) for each of the four categories for the left and right FFA. The boxplots should be read the same way as **a.** arb. units indicate arbitrary units. Source data are provided as a Source Data file

e-f. Same as **a.** but for the EBA and the PPA. Source data are provided as a Source Data file

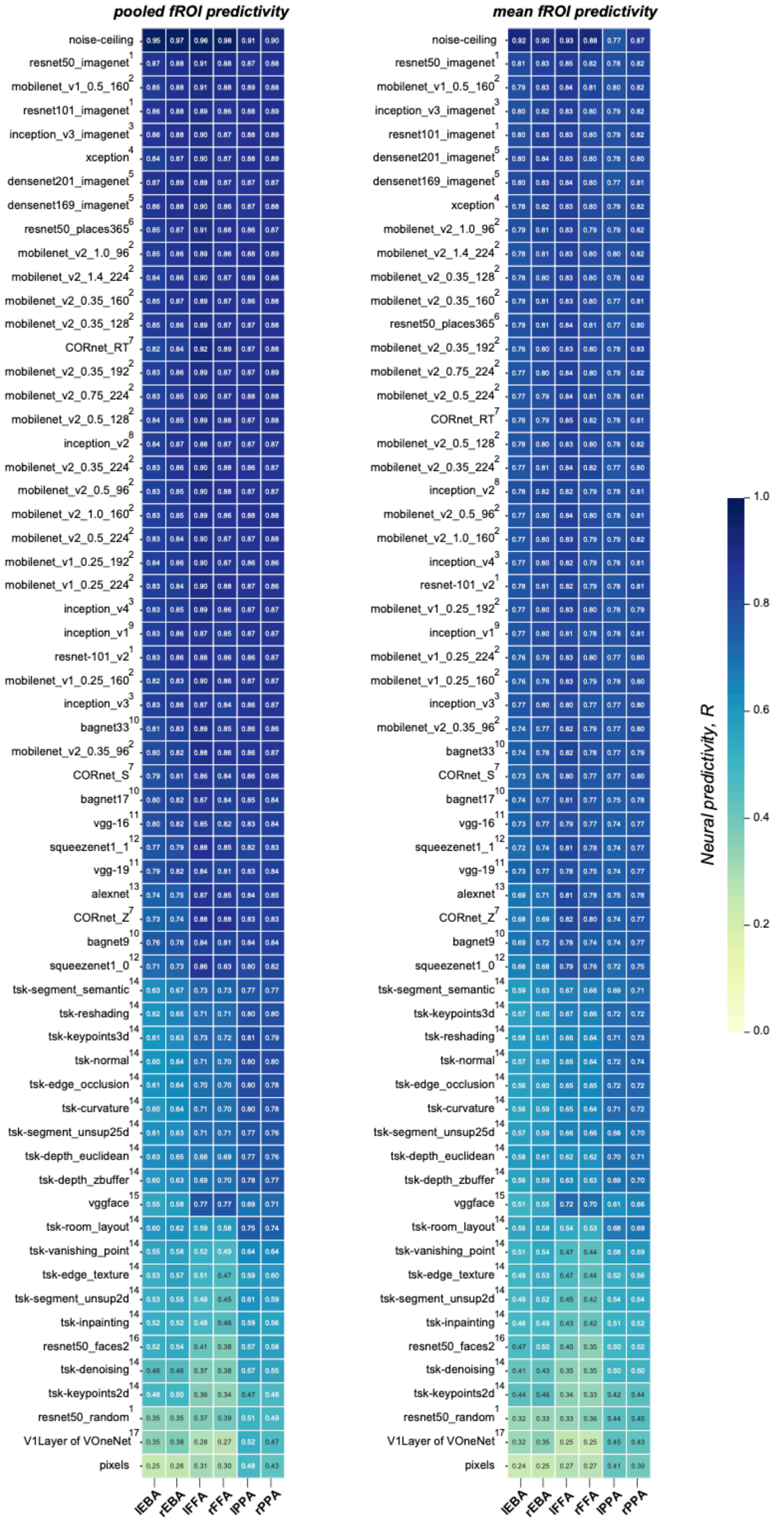

Supplementary Figure 3. Screening computational models. Colormaps showing the performance of 60 computational models (see **Table S1**) at predicting the pooled response across subjects (left), and the mean fROI response in individual subjects (averaged across models) to stimuli in the FFA, PPA, and the EBA. The ordering of models was determined by averaging across the fROIs and sorting the rows from best (top) to the worst (bottom) models individually for each colormap to indicate the consistency of these metrics across scales. Source data are provided as a Source Data file

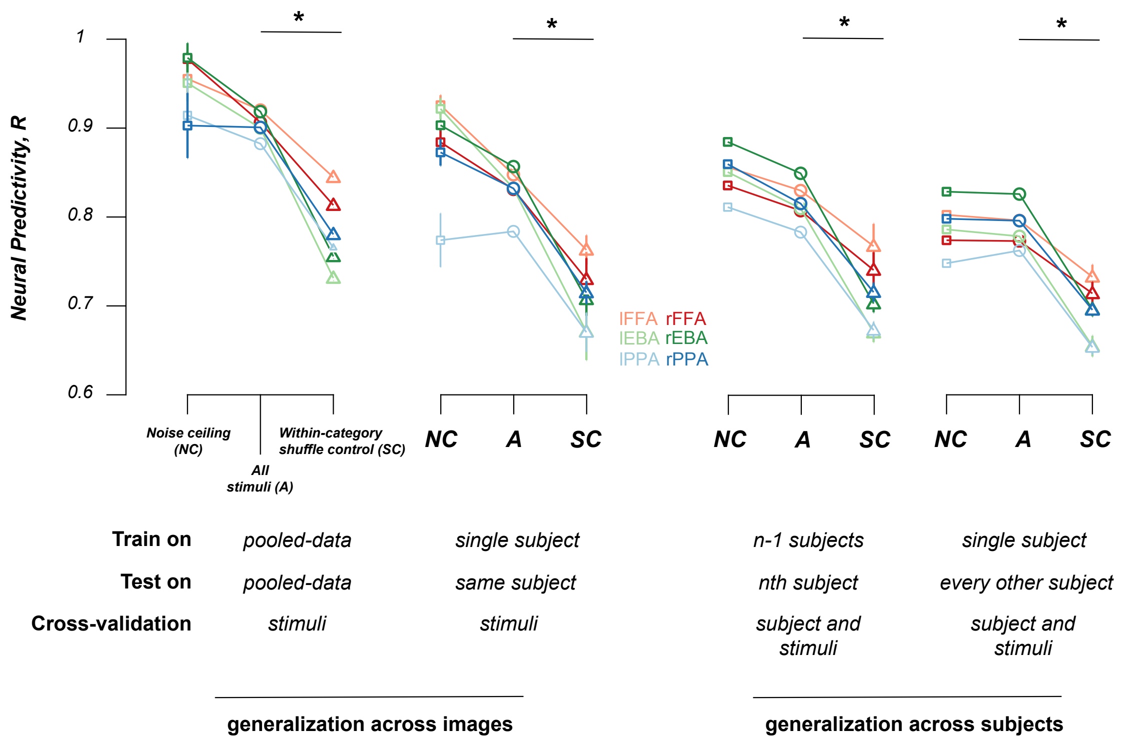

Supplementary Figure 4. DNN-based models generalize across stimuli and subjects. Models were additionally evaluated either by cross-validating across images (columns 1 and 2, left) or on a more stringent criteria across both images and participants (columns 2 and 4, right). In each case, the observed model predictions (circles) were significantly higher than the within-category shuffled controls (triangles) indicating that the models could predict image-level variance over and above the category-level variance. The square markers indicate the estimates of the noise-ceiling (NC) of the data. N = 6 fROIs per comparison. * is $P = 0.03$, two-sided Wilcoxon signed rank test across fROIs between the prediction and the within-category shuffled control, the markers indicate the mean noise ceiling of the data (column 1), the mean prediction accuracy for each fROI (indicated using the colors) and the mean within-category shuffle control estimates. The error bars indicate the standard deviation over 10 random splits of the data (column 1), the standard deviation over subjects in case of generalization over subjects (column 2) and the standard deviation over 50 random shuffles of the data needed to obtain the shuffle control estimates (column 3)

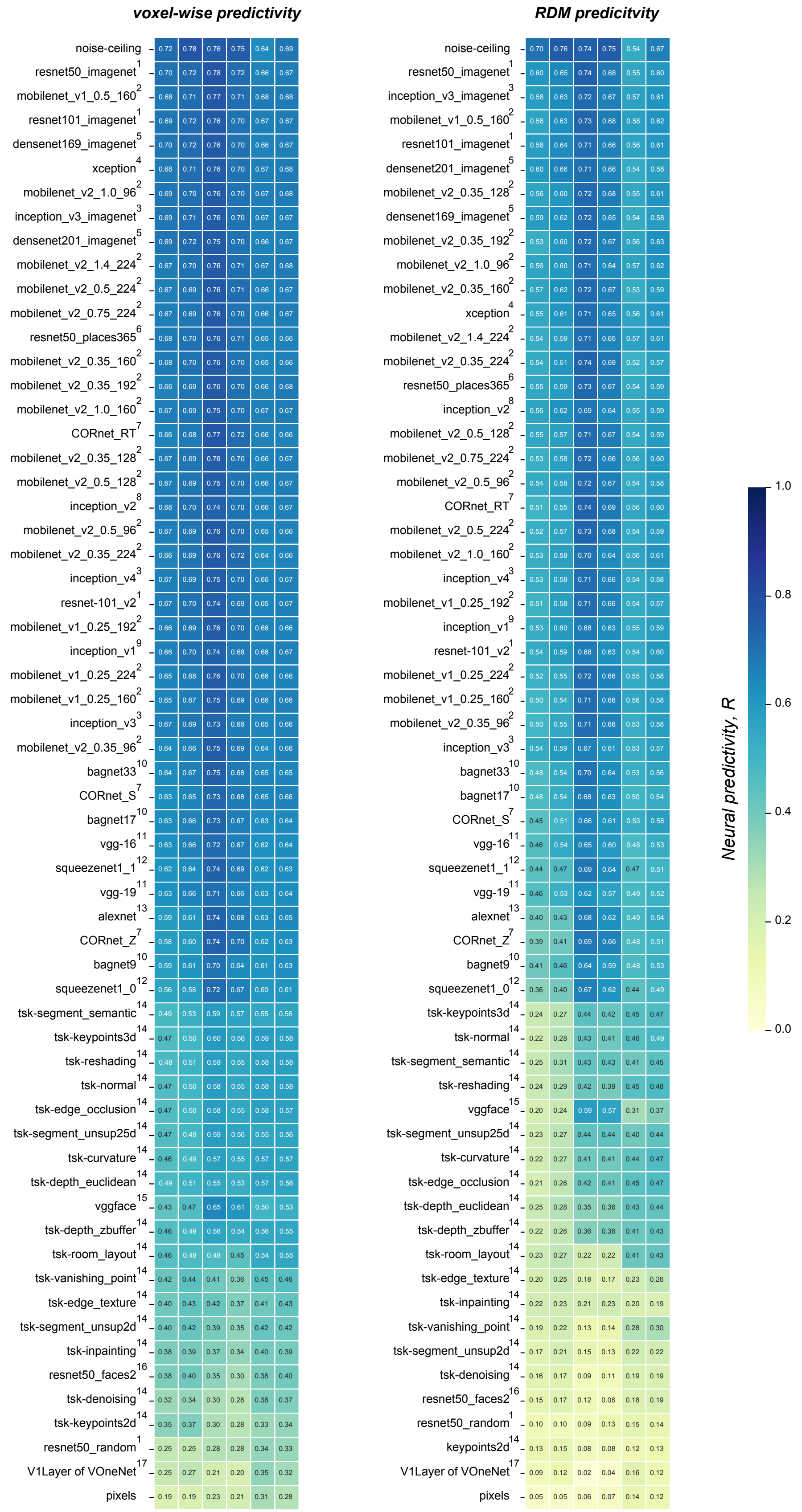

Supplementary Figure 5. Screening computational models. Colormaps showing the mean predictivity of the 60 computational models as before at predicting the voxel-wise response (averaged across participants, left), or the representational dissimilarity (in each subject and then averaged across subjects) in the FFA, PPA, and the EBA. The ordering of models was determined by averaging across the fROIs and sorting the rows from best (top) to the worst (bottom) models individually for each colormap. Source data are provided as a Source Data file

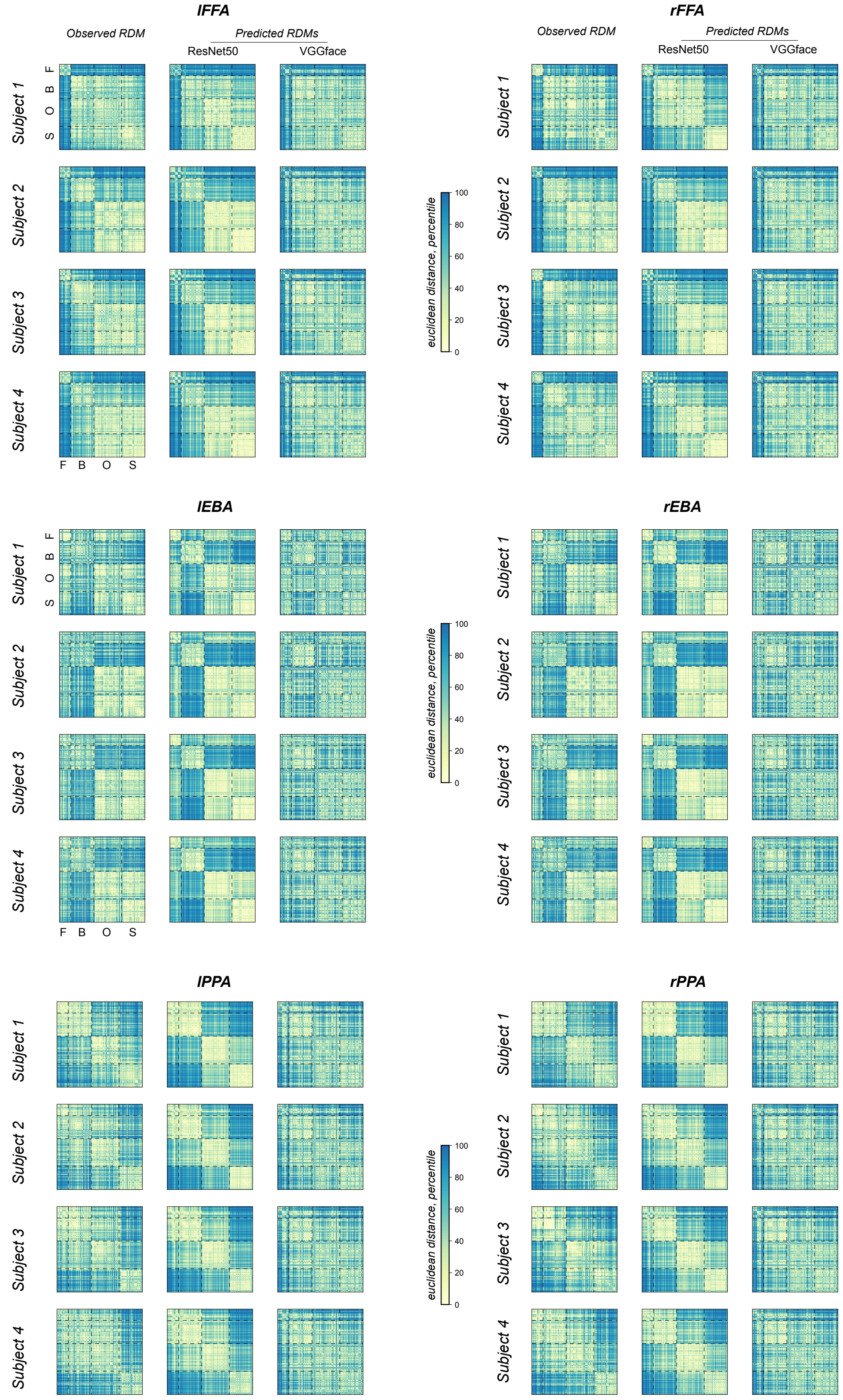

Supplementary Figure 6. Observed and model-predicted RDMs (for 2 representative models) for each subject and fROI. Source data are provided as a Source Data file

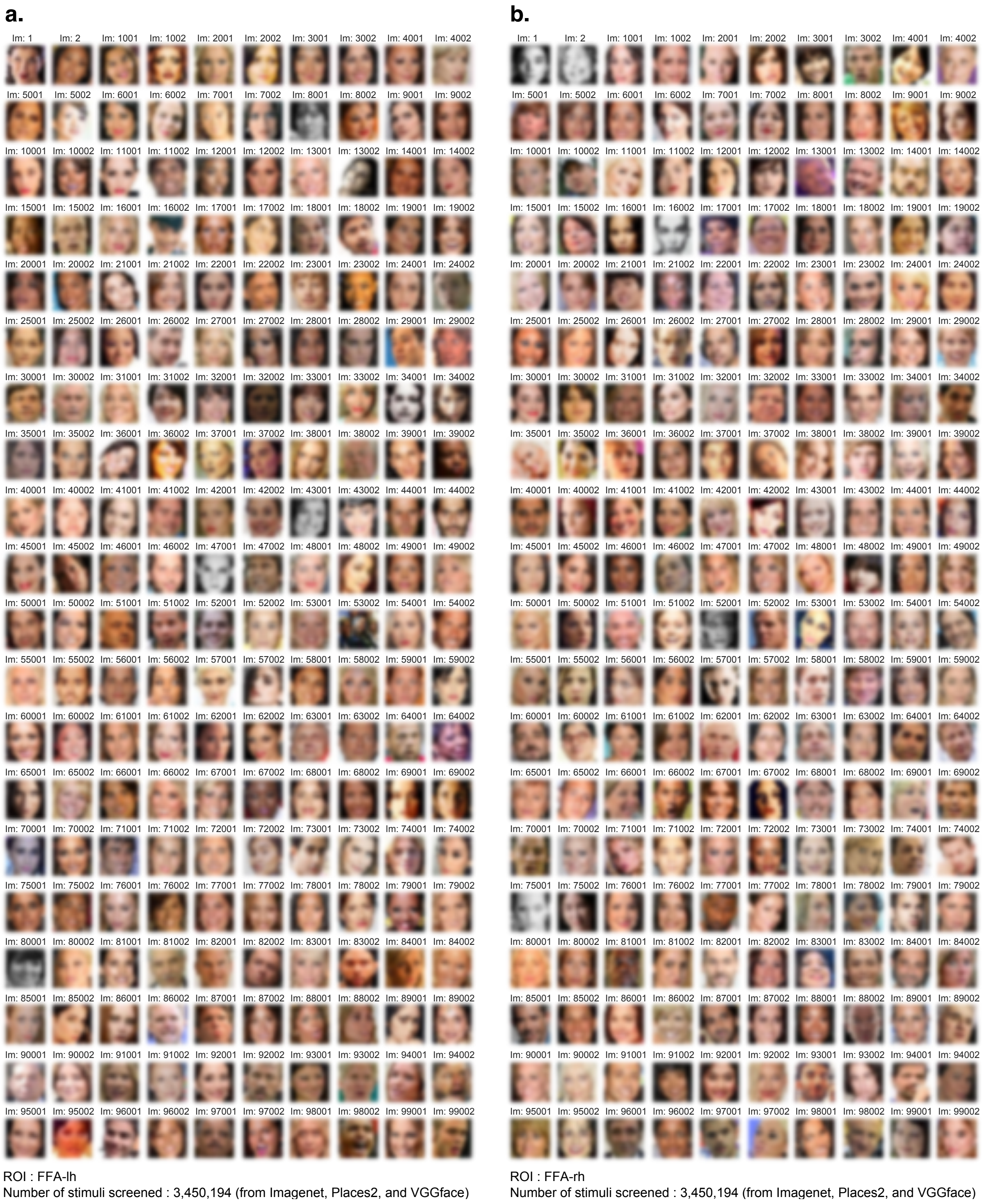

Supplementary Figure 7. Sub-sampled stimuli from the top 100,000 images that the models predict most strongly activate the human a. left FFA and b. right FFA. Note that the images have been blurred to obscure the identity. Original images can be found at https://osf.io/5k4ds/

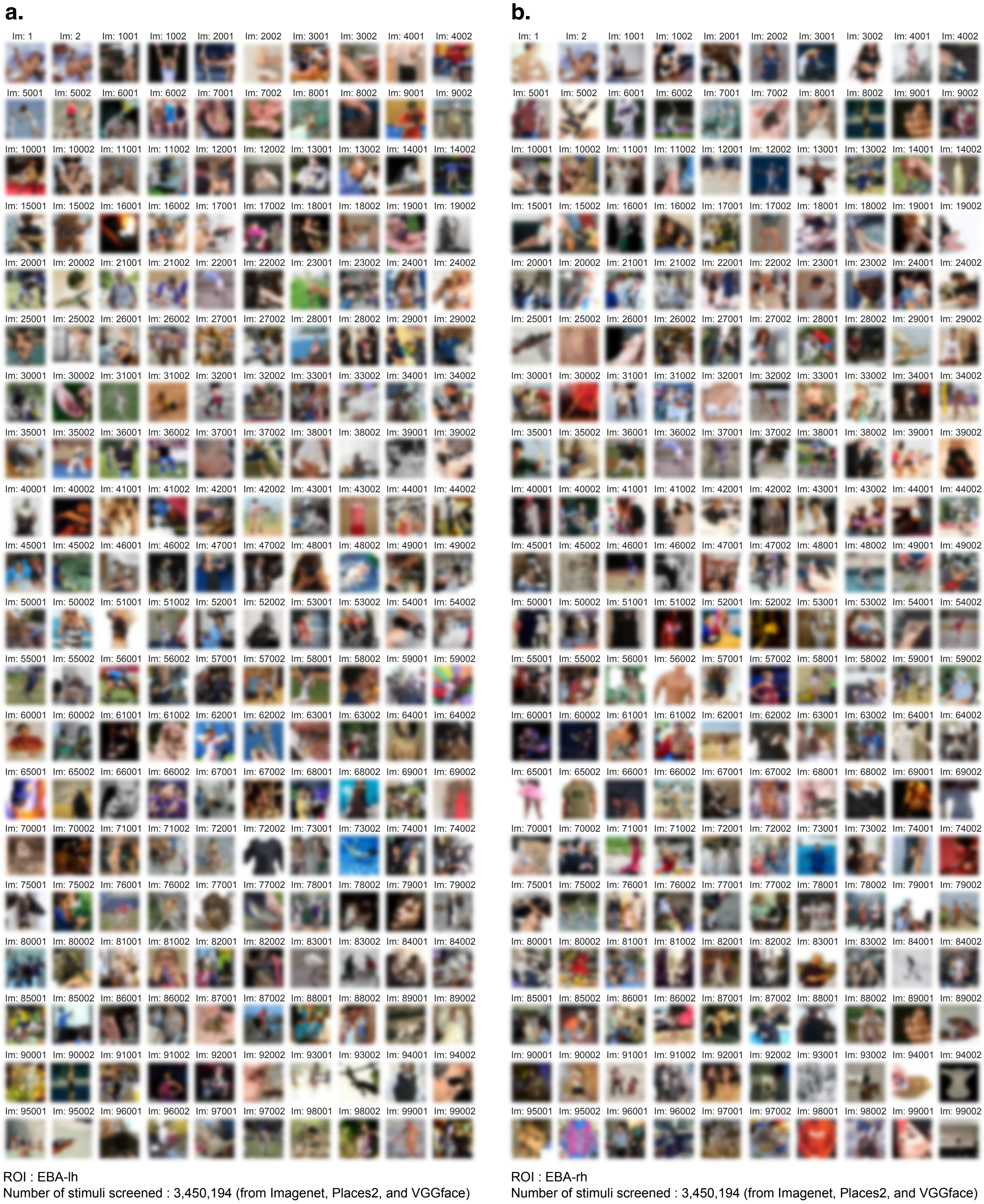

Supplementary Figure 8. Sub-sampled stimuli from the top 100,000 images that the models predict most strongly activate the human a. left EBA and b. right EBA. Note that the images have been blurred to obscure the identity. Original images can be found at https://osf.io/5k4ds/

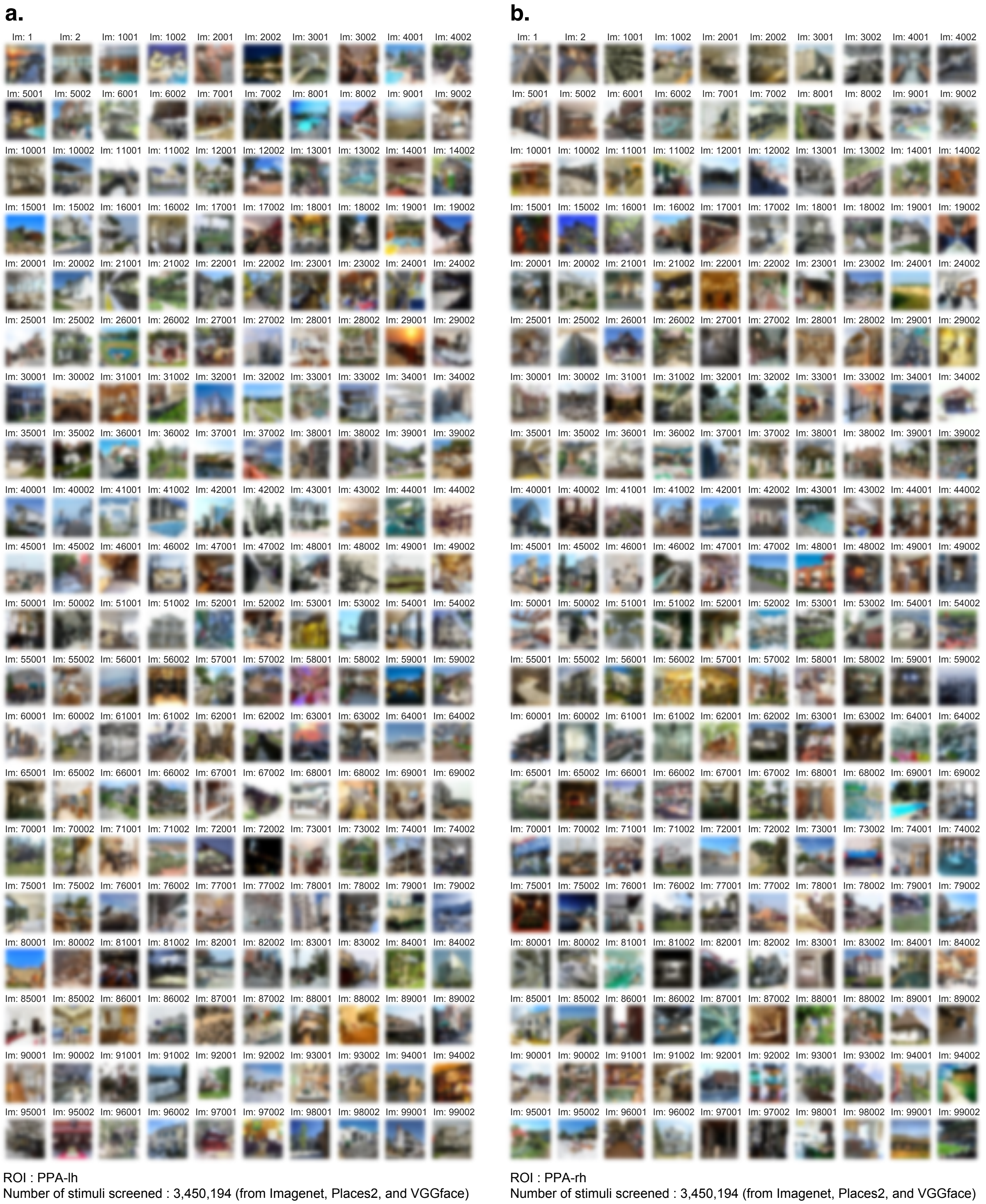

Supplementary Figure 9. Sub-sampled stimuli from the top 100,000 images that the models predict most strongly activate the human a. left PPA and b. right PPA. Note that the images have been blurred to obscure the identity. Original images can be found at https://osf.io/5k4ds/

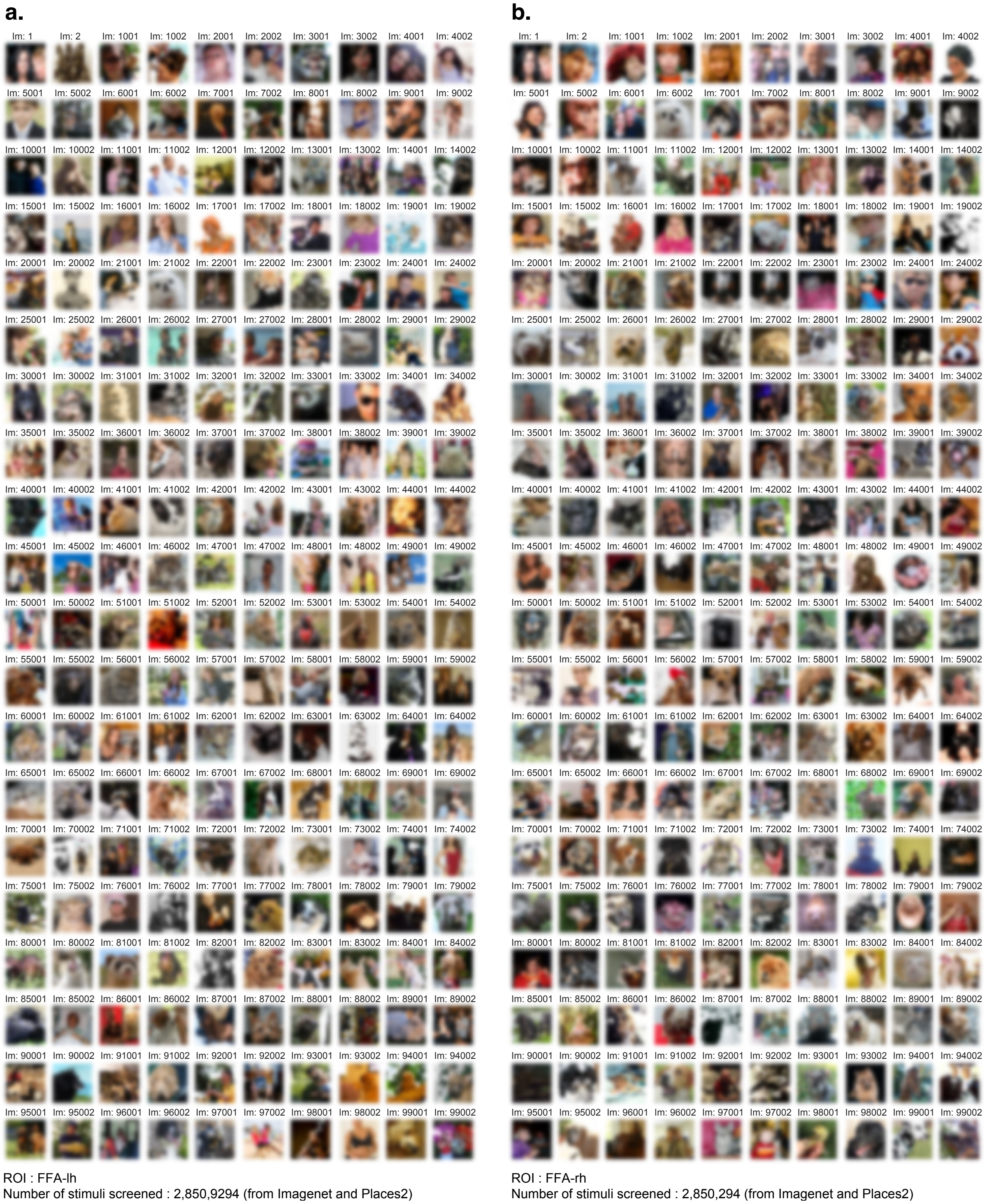

Supplementary Figure 10. Sub-sampled stimuli from the top 100,000 images (excluding stimuli from the VGGFace database) that the models predict most strongly activate the human a. left FFA and b. right FFA. Note that the images have been blurred to obscure the identity. Original images can be found at https://osf.io/5k4ds/

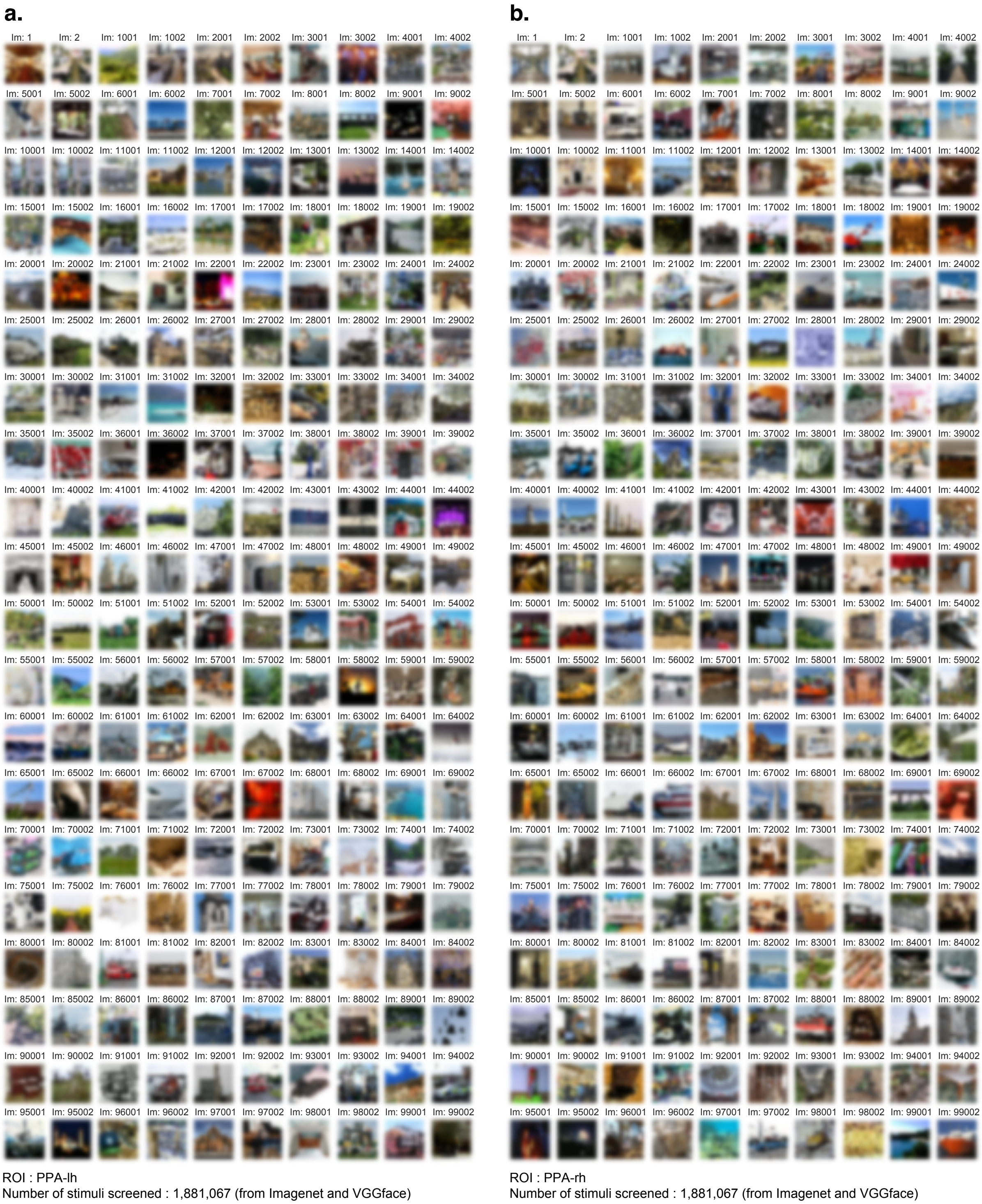

Supplementary Figure 11. Sub-sampled stimuli from the top 100,000 images (excluding stimuli from the Places2 database) that the models predict most strongly activate the human a. left PPA and b. right PPA. Note that the images have been blurred to obscure the identity of the images. Original images can be found at https://osf.io/5k4ds/

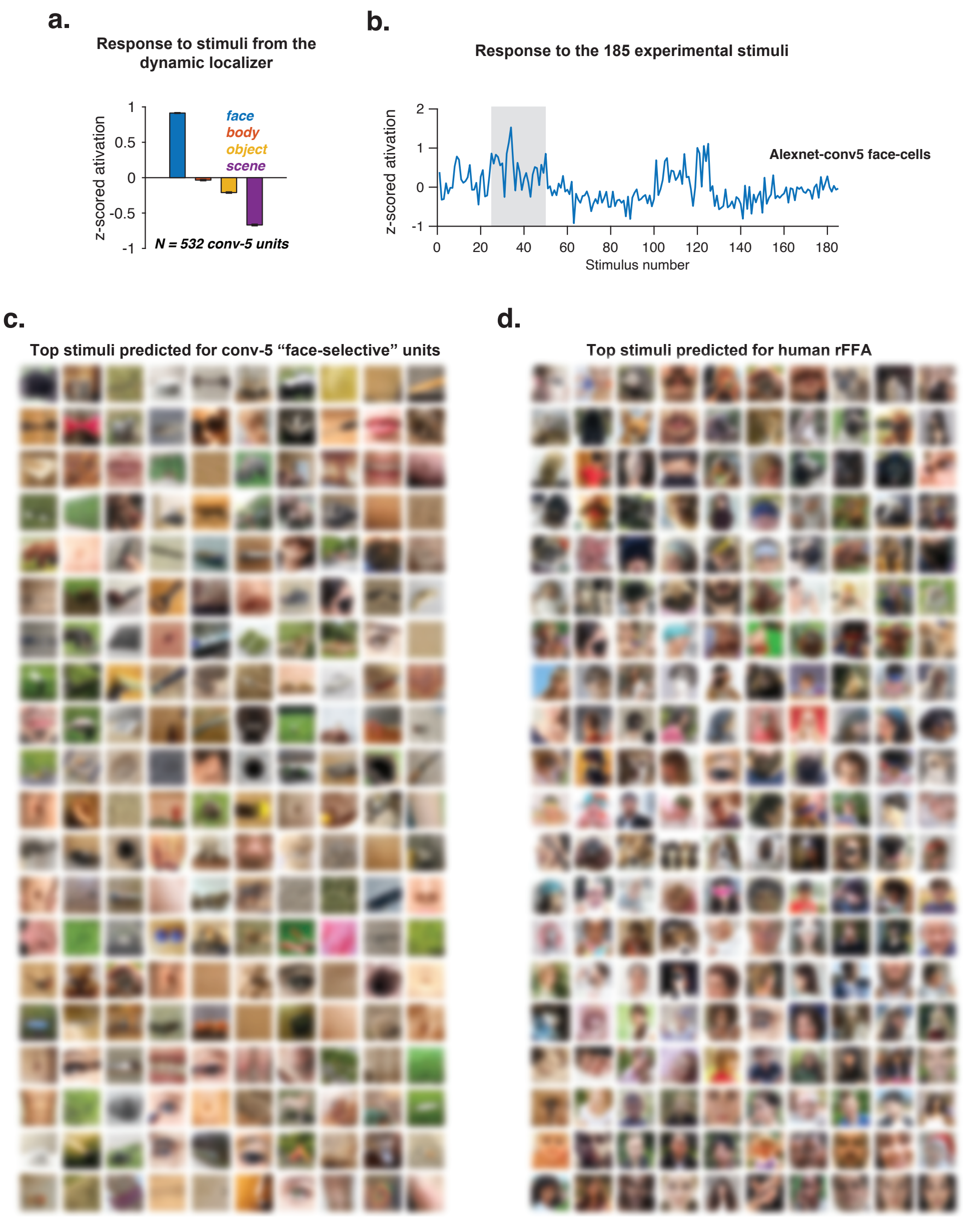

Supplementary Figure 12. Negative control analysis based on simulating the fMRI experiment on conv-5 units of Alexnet

Here we simulated out fMRI experiment in an ANN model to ask whether it is in principle possible that units that met our criteria for face selectivity from our localizer experiment could ever fail to show face selectivity when tested with a broader set of stimuli

a. Face-selective units in Alexnet conv-5 were selected based on a statistical threshold of faces > [bodies, scenes and objects] using snapshots from the localizer videos used in the human experiment at $P<0.000000001$ (uncorrected). The bars show the response profile of these putative face-selective cells in Alexnet to these localizer stimuli for each of the four categories (Note that these responses are not independent, see **b.**). Source data are provided as a Source Data file

b. Response of the putative face selective cells identified from (**a.**) to each of the 185 images used in our experiment. The gray shaded region indicates the face images in the set. This response profile was next modeled using a Resnet-50 (as in the human fMRI experiment). Source data are provided as a Source Data file

c. We next screened stimuli from the THINGS database (N = 27,000 images). The top 200 stimuli that strongly activate the Alexnet conv-5 putative face cells are displayed. Note that only 15/200 (15%are faces). Note that the images have been blurred to obscure the identity. Original images can be found at https://osf.io/5k4ds/

d. The top images that strongly activate the human FFA and plotted next for comparison. Note that the images have been blurred to obscure the identity. Original images can be found at https://osf.io/5k4ds/

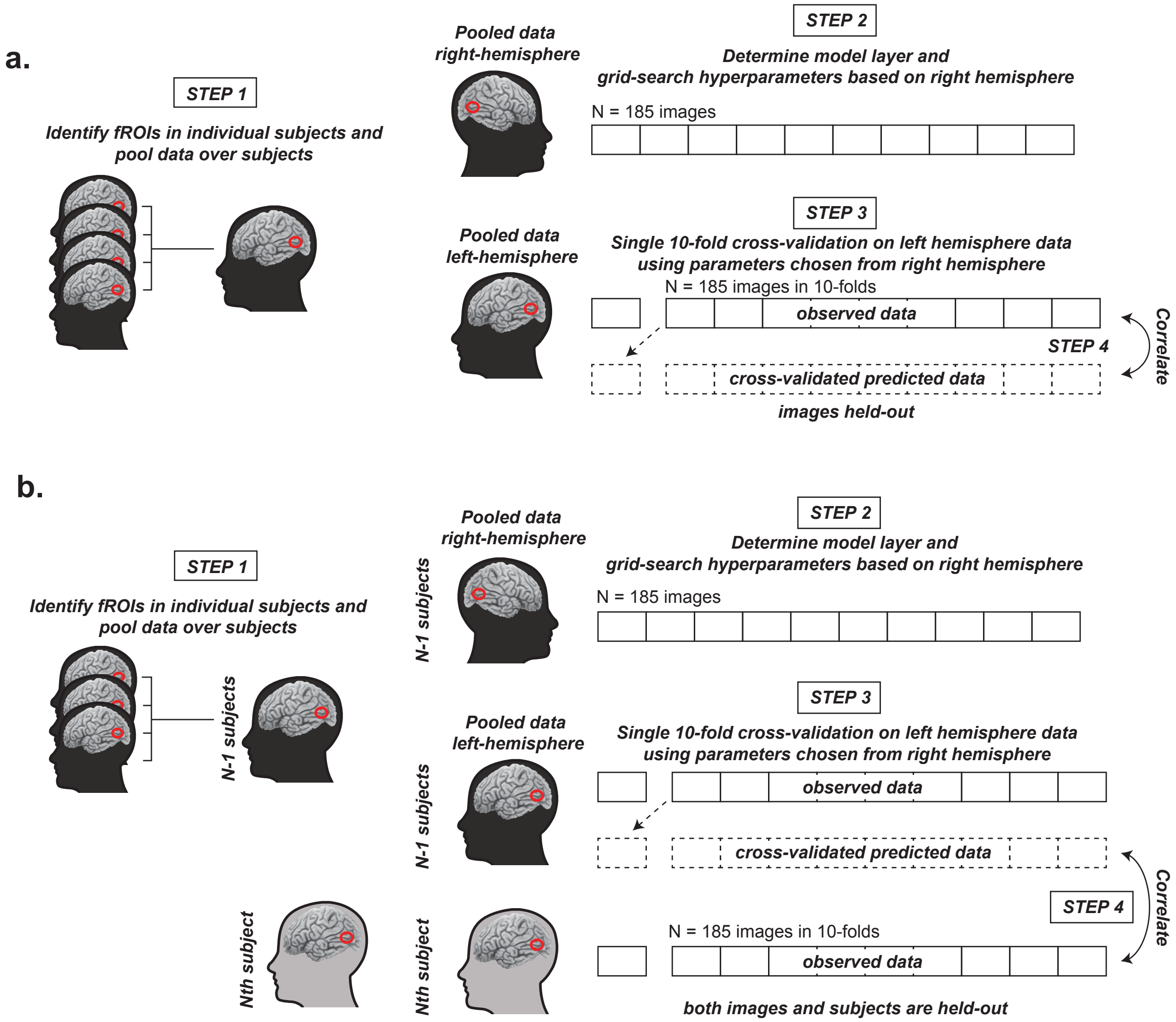

Supplementary Figure 13. Schematic outline of the data-handling procedures for an example region (left-EBA)
a. Pooled predictivity. Here we build models to predict responses of each region to each image, based on data from all subjects, while cross validating over images, and deciding on model parameters based on distinct neural data from the homologous region of the opposite hemisphere. Specifically, in *Step 1*, we pool the fMRI responses over all 4 participants by functionally localizing the region of interest in each participant individually, extracting the magnitude of response of that region to each of the 185 images, and then averaging those response magnitudes across the four participants. The resulting responses to each of the 185 images in that region are the data used to build the model. In *Step 2* we chose all the parameters (which model layer to use and the grid-search hyperparameters) as those that maximize prediction accuracy when this same analysis is run on the distinct neural data from the homologous region of the opposite hemisphere (right-EBA in this case). We then freeze these parameter choices. In *Step 3* we predict data for the left-EBA using a model-mapping procedure with a single 10-fold cross-validation. Importantly, the image whose response magnitude is being predicted is never included in the model-mapping stage. Finally in *Step 4*, we take the Pearson correlation across images between the predicted response to that image from the model and the observed response. This procedure is repeated for every fROI, always using the distinct data from the opposite-hemisphere homologue of that fROI to lock down the layer and grid-search hyperparameters.
b. N-1 predictivity. Here we build models to predict responses in each region to each image, cross validating across images and participants. In *Step 1*, we pool the fMRI responses for the left-EBA over N-1 participants, leaving one subject (Nth subject) out entirely. In *Step 2* we decide the free choices (which model layer to use and the grid-search hyperparameters). These decisions are again based on neural data from the opposite hemisphere (right-EBA in this case). *Step 3* is the same as **a.** where the predicted responses for the right-EBA is obtained using a single 10-fold cross-validation (but note this time based on N-1 subjects' data). Importantly, the image whose response magnitude is being predicted is never included in the model-mapping stage. Finally in *Step 4*, we take the Pearson correlation between the predicted data from Step 3 and the held-out Nth subject's observed data. This entire procedure is then repeated for each subject and fROI. This is the analysis performed to compare models with humans (experts and novices in **Fig. 3**).

MODEL	CITATION	lEBA	rEBA	lFFA	rFFA	lPPA	rPPA
resnet50_imagenet	He et al., 2015[85]	layer4-1-conv2	layer4-1-conv2	layer4-1-conv2	layer4-1-conv2	layer3-5-conv2	layer3-5-conv2
mobilenet_v1_0.5_160	Howard et al., 2017[86]	Conv2d_13_pointwise	AvgPool_1a	Conv2d_13_depthwise	Conv2d_13_depthwise	Conv2d_13_pointwise	Conv2d_13_pointwise
resnet101_imagenet	He et al., 2015[85]	layer3-22-conv2	layer3-22-conv2	layer3-22-conv2	layer3-16-conv2	layer3-22-conv2	layer3-22-conv2
inception_v3_imagenet	Szegedy et al., 2015 [87]	Mixed_7b-branch3x3dbl_2-conv	Mixed_7b-branch3x3dbl_2-conv	Mixed_6d-branch7x7dbl_5-conv	Mixed_6d-branch7x7dbl_3-conv	Mixed_7b-branch_pool-conv	Mixed_7b-branch_pool-conv
xception	Chollet et al., 2016 [88]	block11_sepconv2_act	block11_sepconv2_act	block10_sepconv2_act	block10_sepconv2_act	block10_sepconv2_act	block10_sepconv2_act
densenet201_imagenet	Huang et al., 2016 [89]	block4-denselayer2-conv2	denseblock4-denselayer2-conv2	block3-denselayer40-conv1	block3-denselayer40-conv1	block4-denselayer2-conv1	block4-denselayer2-conv1
densenet169_imagenet	Huang et al., 2016 [89]	block4-denselayer5-conv1	denseblock4-denselayer5-conv1	block4-denselayer5-conv1	block4-denselayer5-conv1	block4-denselayer5-conv1	block4-denselayer5-conv1
resnet50_places365	Zhou et al., 2017 [90]	layer4-1-conv2	layer4-1-conv2	layer4-1-conv2	layer4-1-conv2	layer4-1-conv2	layer4-1-conv2
mobilenet_v2_1.0_96	Howard et al., 2017[86]	layer_16/output	layer_16/output	layer_16/output	layer_16/output	layer_16/output	layer_16/output
mobilenet_v2_0.35_160	Howard et al., 2017[86]	global_pool	global_pool	layer_18/output	layer_16/output	layer_18/output	layer_18/output
mobilenet_v2_0.35_128	Howard et al., 2017[86]	global_pool	global_pool	layer_18/output	layer_17/output	global_pool	layer_18/output
CORnet_RT	Kubilius et al., 2018 [91]	IT	IT	IT	IT	IT	IT
mobilenet_v2_0.35_192	Howard et al., 2017[86]	global_pool	global_pool	layer_18/output	layer_16/output	layer_18/output	layer_18/output
mobilenet_v2_0.75_224	Howard et al., 2017[86]	layer_16/output	layer_16/output	layer_16/output	layer_16/output	layer_16/output	layer_16/output
mobilenet_v2_0.5_128	Howard et al., 2017[86]	layer_17/output	layer_17/output	layer_15/output	layer_15/output	layer_16/output	layer_16/output
inception_v2	Szegedy et al., 2015 [92]	Mixed_5b	Mixed_5b	Mixed_5a	Mixed_5a	Mixed_5a	Mixed_5a
mobilenet_v2_0.35_224	Howard et al., 2017[86]	global_pool	global_pool	layer_18/output	layer_16/output	layer_17/output	layer_18/output
mobilenet_v2_0.5_96	Howard et al., 2017[86]	layer_17/output	layer_18/output	layer_16/output	layer_16/output	layer_17/output	layer_17/output
mobilenet_v2_1.0_160	Howard et al., 2017[86]	layer_16/output	layer_16/output	layer_16/output	layer_15/output	layer_16/output	layer_15/output
mobilenet_v2_0.5_224	Howard et al., 2017[86]	layer_17/output	layer_17/output	layer_16/output	layer_16/output	layer_17/output	layer_17/output
mobilenet_v1_0.25_192	Howard et al., 2017[86]	AvgPool_1a	AvgPool_1a	Conv2d_13_depthwise	Conv2d_13_depthwise	Conv2d_13_pointwise	Conv2d_13_depthwise
mobilenet_v1_0.25_224	Howard et al., 2017[86]	AvgPool_1a	AvgPool_1a	Conv2d_13_depthwise	Conv2d_13_depthwise	Conv2d_13_pointwise	Conv2d_13_pointwise
inception_v4	Szegedy et al., 2016 [87]	Mixed_7b	Mixed_6e	Mixed_6b	Mixed_6b	Mixed_6d	Mixed_6b
inception_v1	Szegedy et al., 2014 [93]	Mixed_5b	Mixed_5b	Mixed_4e	Mixed_4e	Mixed_5b	Mixed_5b
resnet-101_v2	He et al., 2015[85]	block4/unit_2/bottleneck_v2	block4/unit_2/bottleneck_v2	block4/unit_2/bottleneck_v2	block4/unit_2/bottleneck_v2	block4/unit_1/bottleneck_v2	block4/unit_1/bottleneck_v2
mobilenet_v1_0.25_160	Howard et al., 2017[86]	AvgPool_1a	AvgPool_1a	Conv2d_13_pointwise	Conv2d_13_depthwise	Conv2d_13_pointwise	Conv2d_13_pointwise
inception_v3	Szegedy et al., 2015 [87]	Mixed_7b	Mixed_7a	Mixed_7a	Mixed_6d	Mixed_7a	Mixed_7a
bagnet33	Brendel et al., 2019 [94]	avgpool	avgpool	avgpool	avgpool	avgpool	avgpool
mobilenet_v2_0.35_96	Howard et al., 2017[86]	global_pool	global_pool	layer_18/output	layer_17/output	layer_16/output	layer_17/output
CORnet_S	Kubilius et al., 2018 [91]	V4	IT	V4	V4	IT	V4
bagnet17	Brendel et al., 2019 [94]	avgpool	avgpool	avgpool	avgpool	avgpool	avgpool
vgg-16	Simonyan et al., 2014 [95]	fc2	fc2	block5_pool	block4_pool	fc2	fc2
squeezenet1_1	Iandola et al., 2016 [96]	f.11.expand3x3_activation	f.11.expand3x3_activation	f.11.expand3x3_activation	f.11.expand3x3_activation	f.11.expand3x3_activation	f.11.expand3x3_activation
vgg-19	Simonyan et al., 2014 [95]	fc2	fc2	block5_pool	block5_pool	fc2	fc2

alexnet	Krizhevsky et al., 2012 [97]	features_10	features_10	features_10	features_10	features_10	features_10
CORnet_Z	Kubilius et al., 2018 [91]	IT	IT	IT	IT	IT	IT
bagnet9	Brendel et al., 2019 [94]	avgpool	avgpool	avgpool	avgpool	avgpool	avgpool
squeezenet1_0	Iandola et al., 2016 [96]	f.10.expand3x3_activation	f.10.expand3x3_activation	f.10.expand3x3_activation	f.10.expand3x3_activation	f.12.expand3x3_activation	f.12.expand3x3_activation
segment_semantic	Zamir et al., 2018 [98]	layer2-2-conv3	layer4-0-downsample-0	layer3-5-conv1	layer3-5-conv1	layer3-5-conv1	layer3-5-conv1
reshading	Zamir et al., 2018 [98]	layer3-0-conv1	layer3-1-conv3	layer4-1-conv1	layer4-0-conv1	layer4-2-conv1	layer4-2-conv1
keypoints3d	Zamir et al., 2018 [98]	layer3-0-conv2	layer3-0-conv2	layer4-0-conv1	layer4-0-conv1	layer4-0-conv2	layer3-4-conv1
normal	Zamir et al., 2018 [98]	layer2-1-conv2	layer2-1-conv2	layer3-4-conv1	layer3-4-conv1	layer4-2-conv2	layer4-2-conv2
edge_occlusion	Zamir et al., 2018 [98]	layer3-0-conv3	layer3-0-conv3	layer2-2-conv2	layer4-0-conv2	layer4-1-conv1	layer4-1-conv1
curvature	Zamir et al., 2018 [98]	layer4-0-conv2	layer3-1-conv2	layer3-1-conv2	layer3-1-conv2	layer4-0-conv2	layer4-0-conv2
segment_unsup25d	Zamir et al., 2018 [98]	layer3-1-conv3	layer3-2-conv1	layer3-3-conv2	layer3-3-conv2	layer3-4-conv1	layer3-4-conv1
depth_euclidean	Zamir et al., 2018 [98]	layer2-1-conv2	layer2-1-conv2	layer4-0-conv2	layer4-0-conv2	layer3-0-conv1	layer3-0-conv1
depth_zbuffer	Zamir et al., 2018 [98]	layer3-1-conv3	layer2-2-conv2	layer4-0-conv1	layer4-0-conv1	layer3-4-conv1	layer4-0-conv1
vggface	Parkhi et al., 2015 [99]	fc1	block5_pool	block5_pool	block5_pool	block5_pool	block5_pool
room_layout	Zamir et al., 2018 [98]	layer2-3-conv2	layer2-3-conv2	layer3-5-conv1	layer3-5-conv1	layer4-0-downsample-0	layer4-0-downsample-0
vanishing_point	Zamir et al., 2018 [98]	layer1-0-conv3	layer1-0-conv2	layer4-1-conv2	layer4-0-conv2	layer4-1-conv2	layer4-1-conv2
edge_texture	Zamir et al., 2018 [98]	layer3-0-conv2	layer2-1-conv3	layer4-0-conv2	layer4-0-conv2	layer4-0-conv2	layer4-0-conv2
segment_unsup2d	Zamir et al., 2018 [98]	layer2-3-conv1	layer2-3-conv1	layer4-0-conv1	layer4-0-conv1	layer4-0-conv2	layer3-0-conv2
inpainting	Zamir et al., 2018 [98]	layer4-0-conv2	layer4-0-conv2	layer4-0-conv2	layer4-0-conv2	layer1-2-conv1	layer1-0-conv2
resnet50_faces2	Cao et al., 2018 [100]	layer1-0-conv2	layer1-0-conv2	layer4-0-conv3	layer4-1-conv1	layer1-0-conv2	layer1-0-conv2
denoising	Zamir et al., 2018 [98]	layer2-1-conv1	layer2-1-conv1	layer1-0-downsample-0	layer1-1-conv1	layer1-2-conv1	layer1-2-conv1
keypoints2d	Zamir et al., 2018 [98]	layer4-0-conv1	layer4-0-conv1	layer4-0-conv2	layer4-0-conv2	layer4-0-conv2	layer4-0-conv1
resnet50_random	He et al., 2015 [85]	layer1-0-downsample-0	layer1-0-downsample-0	layer1-0-conv2	layer1-0-conv2	layer1-0-conv2	layer1-0-conv2
VOneNet_resnet50	Dapello et al., 2020 [101]	VOne_block	VOne_block	VOne_block	VOne_block	VOne_block	VOne_block
pixels		pixels	pixels	pixels	pixels	pixels	pixels

Supplementary Table 1. Summary of all the models evaluated in the current study with the model layer with the best cross-validated accuracy at predicting the responses to images in the different fROIs

References –

1. Kanwisher, N., McDermott, J. & Chun, M. M. The fusiform face area: a module in human extrastriate cortex specialized for face perception. *J. Neurosci.* **17**, 4302–11 (1997).
2. Epstein, R. & Kanwisher, N. A cortical representation the local visual environment. *Nature* **392**, 598–601 (1998).
3. Downing, P. & Kanwisher, N. A cortical area specialized for visual processing of the human body. *Science (80-.).* **293**, 2470–2473 (2001).
4. Powell, L. J., Kosakowski, H. L. & Saxe, R. Social Origins of Cortical Face Areas. *Trends in Cognitive Sciences* vol. 22 752–763 (2018).
5. Deen, B. *et al.* Organization of high-level visual cortex in human infants. *Nat. Commun.* **8**, (2017).
6. Peelen, M. V. & Downing, P. E. Category selectivity in human visual cortex: Beyond visual object recognition. *Neuropsychologia* **105**, 177–183 (2017).
7. Op de Beeck, H. P., Pillet, I. & Ritchie, J. B. Factors Determining Where Category-Selective Areas Emerge in Visual Cortex. *Trends Cogn. Sci.* **0**, (2019).
8. Grill-Spector, K., Weiner, K. S., Kay, K. & Gomez, J. The Functional Neuroanatomy of Human Face Perception. *Annual Review of Vision Science* vol. 3 167–196 (2017).
9. Duchaine, B. & Yovel, G. A revised neural framework for face processing. *Annu. Rev. Vis. Sci.* **1**, 393–416 (2015).
10. Costantini, M., Urgesi, C., Galati, G., Romani, G. L. & Aglioti, S. M. Haptic perception and body representation in lateral and medial occipito-temporal cortices. *Neuropsychologia* **49**, 821–829 (2011).
11. Popivanov, I. D., Schyns, P. G. & Vogels, R. Stimulus features coded by single neurons of a macaque body category selective patch. *Proc. Natl. Acad. Sci.* 201520371 (2016) doi:10.1073/pnas.1520371113.
12. Weiner, K. S. *et al.* The cytoarchitecture of domain-specific regions in human high-level visual cortex. *Cereb. Cortex* **27**, 146–161 (2017).
13. Epstein, R., Harris, A., Stanley, D. & Kanwisher, N. The parahippocampal place area: Recognition, navigation, or encoding? *Neuron* **23**, 115–125 (1999).
14. Epstein, R. A. & Baker, C. I. Scene Perception in the Human Brain. *Annu. Rev. Vis. Sci.* **5**, 373–397 (2019).
15. Krizhevsky, A., Sutskever, I. & Hinton, G. E. ImageNet classification with deep convolutional neural networks. *Commun. ACM* **60**, 84–90 (2017).
16. LeCun, Y., Bengio, Y. & Hinton, G. Deep learning. *Nature* vol. 521 436–444 (2015).
17. Yosinski, J., Clune, J., Nguyen, A., Fuchs, T. & Lipson, H. Understanding Neural Networks Through Deep Visualization. *arXiv* (2015).
18. Nguyen, A., Yosinski, J. & Clune, J. Multifaceted Feature Visualization: Uncovering the Different Types of Features Learned By Each Neuron in Deep Neural Networks. *arXiv* (2016).
19. Wen, H., Shi, J., Chen, W. & Liu, Z. Visualized layer-wise visual features in deep residual neural network. in *Purdue University Research Repository* (2017). doi:10.4231/R7PR7T1G.
20. Yamins, D. L. K. *et al.* Performance-optimized hierarchical models predict neural responses in higher visual cortex. *Proc. Natl. Acad. Sci.* **111**, 8619–8624 (2014).
21. Schrimpf, M. *et al.* Brain-Score: Which Artificial Neural Network for Object Recognition is most Brain-Like? *bioRxiv* 407007 (2018) doi:10.1101/407007.
22. Güçlü, U. & van Gerven, M. A. J. Deep neural networks reveal a gradient in the

complexity of neural representations across the ventral stream. *J. Neurosci.* **35**, 10005–10014 (2015).
23. Eickenberg, M., Gramfort, A., Varoquaux, G. & Thirion, B. Seeing it all: Convolutional network layers map the function of the human visual system. *Neuroimage* **152**, 184–194 (2017).
24. Kanwisher, N. Functional specificity in the human brain: A window into the functional architecture of the mind. *Proc. Natl. Acad. Sci. U. S. A.* 1–8 (2010) doi:10.1073/pnas.1005062107.
25. Walker, E. Y. *et al.* Inception loops discover what excites neurons most using deep predictive models. *Nat. Neurosci.* **22**, 2060–2065 (2019).
26. Bashivan, P., Kar, K. & DiCarlo, J. J. Neural population control via deep image synthesis. *Science (80-.).* **364**, (2019).
27. Ponce, C. R. *et al.* Evolving Images for Visual Neurons Using a Deep Generative Network Reveals Coding Principles and Neuronal Preferences. *Cell* **177**, 999-1009.e10 (2019).
28. Julian, J. B., Fedorenko, E., Webster, J. & Kanwisher, N. An algorithmic method for functionally defining regions of interest in the ventral visual pathway. *Neuroimage* **60**, 2357–2364 (2012).
29. Murty, N. A. R. *et al.* Visual Experience is not Necessary for the Development of Face Selectivity in the Lateral Fusiform Gyrus. *bioRxiv* 2020.02.25.964890 (2020) doi:10.1101/2020.02.25.964890.
30. Lafer-Sousa, R., Conway, B. R. & Kanwisher, N. G. Color-Biased Regions of the Ventral Visual Pathway Lie between Face- and Place-Selective Regions in Humans, as in Macaques. *J. Neurosci.* **36**, 1682–97 (2016).
31. Huth, A. G., De Heer, W. A., Griffiths, T. L., Theunissen, F. E. & Gallant, J. L. Natural speech reveals the semantic maps that tile human cerebral cortex. *Nature* **532**, 453–458 (2016).
32. Agrawal, P., Stansbury, D., Malik, J. & Gallant, J. L. Pixels to Voxels: Modeling Visual Representation in the Human Brain. (2014).
33. Schrimpf, M. *et al.* Integrative Benchmarking to Advance Neurally Mechanistic Models of Human Intelligence. *Neuron* vol. 108 413–423 (2020).
34. Kubilius, J. *et al.* CORnet: Modeling the Neural Mechanisms of Core Object Recognition. *bioRxiv* 408385 (2018) doi:10.1101/408385.
35. Russakovsky, O. *et al.* ImageNet Large Scale Visual Recognition Challenge. *Int. J. Comput. Vis.* **115**, 211–252 (2015).
36. Zhou, B., Lapedriza, A., Khosla, A., Oliva, A. & Torralba, A. Places: A 10 Million Image Database for Scene Recognition. *IEEE Trans. Pattern Anal. Mach. Intell.* **40**, 1452–1464 (2018).
37. Parkhi, O. M., Vedaldi, A. & Zisserman, A. Deep Face Recognition. in *British Machine Vision Conference* 41.1-41.12 (2015). doi:10.5244/c.29.41.
38. Wen, H., Shi, J., Chen, W. & Liu, Z. Deep Residual Network Predicts Cortical Representation and Organization of Visual Features for Rapid Categorization. *Sci. Rep.* **8**, (2018).
39. Naselaris, T., Kay, K. N., Nishimoto, S. & Gallant, J. L. *Encoding and decoding in fMRI. NeuroImage* vol. 56 (2011).
40. Seeliger, K. *et al.* End-to-end neural system identification with neural information flow. *PLOS Comput. Biol.* **17**, e1008558 (2021).
41. Rips, L. J. . Similarity, typicality, and categorization. in *Similarity and Analogical Reasoning* 21–59 (Cambridge University Press, 2009). doi:10.1017/cbo9780511529863.004.

42. Medin, D. L. Concepts and Conceptual Structure. *Am. Psychol.* **44**, 1469–1481 (1989).
43. Petsiuk, V., Das, A. & Saenko, K. RISE: Randomized input sampling for explanation of black-box models. *Br. Mach. Vis. Conf. 2018, BMVC 2018* **1**, (2019).
44. Hong, H., Yamins, D. L. K., Majaj, N. J. & DiCarlo, J. J. Explicit information for category-orthogonal object properties increases along the ventral stream. *Nat. Neurosci.* (2016) doi:10.1038/nn.4247.
45. Güçlü, U. & van Gerven, M. A. J. Increasingly complex representations of natural movies across the dorsal stream are shared between subjects. *Neuroimage* **145**, 329–336 (2017).
46. Khaligh-Razavi, S.-M. & Kriegeskorte, N. Deep Supervised, but Not Unsupervised, Models May Explain IT Cortical Representation. *PLoS Comput Biol* **10**, 1003915 (2014).
47. Cichy, R. M., Khosla, A., Pantazis, D., Torralba, A. & Oliva, & A. Comparison of deep neural networks to spatio-temporal cortical dynamics of human visual object recognition reveals hierarchical correspondence OPEN. *Nat. Publ. Gr.* (2016) doi:10.1038/srep27755.
48. Tarhan, L. & Konkle, T. Sociality and interaction envelope organize visual action representations. *Nat. Commun.* **11**, (2020).
49. Lee, H. *et al.* Topographic deep artificial neural networks reproduce the hallmarks of the primate inferior temporal cortex face processing network. *bioRxiv Prepr.* (2020) doi:10.1101/2020.07.09.185116.
50. Zhuang, C. *et al.* Unsupervised Neural Network Models of the Ventral Visual Stream. *bioRxiv Prepr.* 2020.06.16.155556 (2020) doi:10.1101/2020.06.16.155556.
51. Konkle, T. & Alvarez, G. A. Instance-level contrastive learning yields human brain-like representation without category-supervision. *bioRxiv Prepr.* 2020.06.15.153247 (2020) doi:10.1101/2020.06.15.153247.
52. Deza, A. & Konkle, T. Emergent Properties of Foveated Perceptual Systems. *bioRxiv Prepr.* (2020).
53. Szegedy, C. *et al.* Intriguing properties of neural networks. in *2nd International Conference on Learning Representations, ICLR 2014 - Conference Track Proceedings* (2014).
54. Goodfellow, I. J., Shlens, J. & Szegedy, C. Explaining and harnessing adversarial examples. in *3rd International Conference on Learning Representations, ICLR 2015 - Conference Track Proceedings* (2015).
55. Downing, P., Liu, J. & Kanwisher, N. Testing cognitive models of visual attention with fMRI and MEG. *Neuropsychologia* **39**, 1329–1342 (2001).
56. Kanwisher, N. & Wojciulik, E. Visual attention: Insights from brain imaging. *Nat. Rev. Neurosci.* **1**, 91–100 (2000).
57. Geirhos, R., Meding, K. & Wichmann, F. A. Beyond accuracy: quantifying trial-by-trial behaviour of CNNs and humans by measuring error consistency. *bioRxiv Prepr.* (2020).
58. Geirhos, R. *et al.* Imagenet-trained CNNs are biased towards texture; increasing shape bias improves accuracy and robustness. in *7th International Conference on Learning Representations, ICLR 2019* (2019).
59. O'Connell, T. P., Sederberg, P. B. & Walther, D. B. Representational differences between line drawings and photographs of natural scenes: A dissociation between multi-voxel pattern analysis and repetition suppression. *Neuropsychologia* **117**, 513–519 (2018).
60. Geirhos, R. *et al.* Generalisation in humans and deep neural networks. in *Advances in Neural Information Processing Systems* vols 2018-Decem 7538–7550 (2018).
61. Dolan, R. J. *et al.* How the brain learns to see objects and faces in an impoverished context. *Nature* **389**, 596–599 (1997).

62. Cox, D., Meyers, E. & Sinha, P. Contextually Evoked Object-Specific Responses in Human Visual Cortex. *Science (80-.).* **304**, 115–117 (2004).
63. Haxby, J. V. *et al.* Distributed and overlapping representations of faces and objects in ventral temporal cortex. *Science (80-.).* **293**, 2425–2430 (2001).
64. Schalk, G. *et al.* Facephenes and rainbows: Causal evidence for functional and anatomical specificity of face and color processing in the human brain. *Proc. Natl. Acad. Sci. U. S. A.* **114**, 12285–12290 (2017).
65. Nguyen, A., Dosovitskiy, A., Yosinski, J., Brox, T. & Clune, J. Synthesizing the preferred inputs for neurons in neural networks via deep generator networks. in *Advances in Neural Information Processing Systems* 3395–3403 (2016).
66. Frankle, J. & Carbin, M. The lottery ticket hypothesis: Finding sparse, trainable neural networks. *arXiv* 1–42 (2018).
67. Yamins, D. L. K. & DiCarlo, J. J. Using goal-driven deep learning models to understand sensory cortex. *Nature Neuroscience* vol. 19 356–365 (2016).
68. Hebart, M. N. *et al.* THINGS: A database of 1,854 object concepts and more than 26,000 naturalistic object images. *PLoS One* **14**, e0223792 (2019).
69. Kay, K. N., Rokem, A., Winawer, J., Dougherty, R. F. & Wandell, B. A. GLMdenoise: A fast, automated technique for denoising task-based fMRI data. *Front. Neurosci.* **7**, (2013).
70. Charest, I., Kriegeskorte, N. & Kay, K. N. GLMdenoise improves multivariate pattern analysis of fMRI data. *Neuroimage* **183**, 606–616 (2018).
71. Kar, K., Kubilius, J., Schmidt, K., Issa, E. B. & DiCarlo, J. J. Evidence that recurrent circuits are critical to the ventral stream's execution of core object recognition behavior. *Nat. Neurosci.* **22**, 974–983 (2019).
72. Naselaris, T., Kay, K. N., Nishimoto, S. & Gallant, J. L. Encoding and decoding in fMRI. *Neuroimage* **56**, 400–410 (2011).
73. Çukur, T., Huth, A. G., Nishimoto, S. & Gallant, J. L. Functional subdomains within scene-selective cortex: Parahippocampal place area, retrosplenial complex, and occipital place area. *J. Neurosci.* **36**, 10257–10273 (2016).
74. Klindt, D. A., Ecker, A. S., Euler, T. & Bethge, M. Neural system identification for large populations separating 'what' and 'where'. in *Advances in Neural Information Processing Systems* vols 2017-Decem 3507–3517 (2017).
75. Davies-Thompson, J. & Andrews, T. J. Intra- and interhemispheric connectivity between face-selective regions in the human brain. *J. Neurophysiol.* **108**, 3087–3095 (2012).
76. Kriegeskorte, N., Mur, M. & Bandettini, P. Representational similarity analysis - connecting the branches of systems neuroscience. *Front. Syst. Neurosci.* **2**, 4 (2008).
77. Mur, M., Bandettini, P. a & Kriegeskorte, N. Revealing representational content with pattern-information fMRI--an introductory guide. *Soc. Cogn. Affect. Neurosci.* **4**, 101–9 (2009).
78. Diedrichsen, J. & Kriegeskorte, N. Representational models: A common framework for understanding encoding, pattern-component, and representational-similarity analysis. *PLoS Comput. Biol.* **13**, (2017).
79. Martin Cichy, R., Roig, G. & Oliva, A. The Algonauts Project. *Nat. Mach. Intell.* doi:10.1038/s42256-019-0127-z.
80. Walther, A. *et al.* Reliability of dissimilarity measures for multi-voxel pattern analysis. *Neuroimage* **137**, 188–200 (2016).
81. Erhan, D., Bengio, Y., Courville, A. & Vincent, P. Visualizing higher-layer features of a deep network. *Univ. Montr.* 1–13 (2009).

82. Goodfellow, I. J. *et al.* Generative adversarial nets. in *Advances in Neural Information Processing Systems* vol. 3 2672–2680 (2014).
83. Brock, A., Donahue, J. & Simonyan, K. Large scale GaN training for high fidelity natural image synthesis. in *7th International Conference on Learning Representations, ICLR 2019* 1–35 (2019).
84. Kingma, D. P. & Ba, J. L. Adam: A method for stochastic optimization. in *3rd International Conference on Learning Representations, ICLR 2015 - Conference Track Proceedings* (2015).
85. He, K., Zhang, X., Ren, S. & Sun, J. Deep residual learning for image recognition. in *Proceedings of the IEEE Computer Society Conference on Computer Vision and Pattern Recognition* vols 2016-Decem 770–778 (2016).
86. Howard, A. G. *et al.* MobileNets: Efficient Convolutional Neural Networks for Mobile Vision Applications. *arXiv* (2017).
87. Szegedy, C., Ioffe, S., Vanhoucke, V. & Alemi, A. A. Inception-v4, inception-ResNet and the impact of residual connections on learning. *31st AAAI Conf. Artif. Intell. AAAI 2017* 4278–4284 (2017).
88. Chollet, F. Xception: Deep Learning with Depthwise Separable Convolutions. in *2017 IEEE Conference on Computer Vision and Pattern Recognition (CVPR)* 1800–1807 (2017). doi:10.1109/CVPR.2017.195.
89. Huang, G., Liu, Z., Van Der Maaten, L. & Weinberger, K. Q. Densely Connected Convolutional Networks. in *2017 IEEE Conference on Computer Vision and Pattern Recognition (CVPR)* 2261–2269 (2017). doi:10.1109/CVPR.2017.243.
90. Zhou, B., Lapedriza, A., Khosla, A., Oliva, A. & Torralba, A. Places: A 10 Million Image Database for Scene Recognition. *IEEE Trans. Pattern Anal. Mach. Intell.* **40**, 1452–1464 (2018).
91. Kubilius, J. *et al.* CORnet: Modeling the Neural Mechanisms of Core Object Recognition. *bioRxiv* 408385 (2018) doi:10.1101/408385.
92. Szegedy, C., Vanhoucke, V., Ioffe, S., Shlens, J. & Wojna, Z. Rethinking the Inception Architecture for Computer Vision. *Proc. IEEE Comput. Soc. Conf. Comput. Vis. Pattern Recognit.* **2016-Decem**, 2818–2826 (2016).
93. Szegedy, C. *et al.* Going deeper with convolutions. *Proc. IEEE Comput. Soc. Conf. Comput. Vis. Pattern Recognit.* **07-12-June**, 1–9 (2015).
94. Brendel, W. & Bethge, M. Approximating cnns with bag-of-local-features models works surprisingly well on Imagenet. *7th Int. Conf. Learn. Represent. ICLR 2019* 1–15 (2019).
95. Simonyan, K. & Zisserman, A. Very deep convolutional networks for large-scale image recognition. *3rd Int. Conf. Learn. Represent. ICLR 2015 - Conf. Track Proc.* 1–14 (2015).
96. Iandola, F. N. *et al.* SqueezeNet: AlexNet-level accuracy with 50x fewer parameters and <0.5MB model size. *arXiv* 1–13 (2016).
97. Krizhevsky, A., Sutskever, I. & Hinton, G. E. ImageNet Classification with Deep Convolutional Neural Networks. in *Advances in Neural Information Processing Systems 25* (eds. Pereira, F., Burges, C. J. C., Bottou, L. & Weinberger, K. Q.) 1097–1105 (Curran Associates, Inc., 2012).
98. Zamir, A. *et al.* Taskonomy: Disentangling task transfer learning. in *IJCAI International Joint Conference on Artificial Intelligence* vols 2019-Augus 6241–6245 (2019).
99. Parkhi, O. M., Vedaldi, A. & Zisserman, A. Deep Face Recognition. *Br. Mach. Vis. Assoc. Soc. Pattern Recognit.* 41.1-41.12 (2015) doi:10.5244/c.29.41.
100. Cao, Q., Shen, L., Xie, W., Parkhi, O. M. & Zisserman, A. VGGFace2: A dataset for recognising faces across pose and age. *Proc. - 13th IEEE Int. Conf. Autom. Face Gesture Recognition, FG 2018* 67–74 (2018) doi:10.1109/FG.2018.00020.

101. Dapello, J. *et al.* Simulating a Primary Visual Cortex at the Front of CNNs Improves Robustness to Image Perturbations. *bioRxiv* 1–26 (2020) doi:10.1101/2020.06.16.154542.
